# Cooperative contributions of structural and functional connectivity to successful memory in aging

**DOI:** 10.1162/netn_a_00064

**Published:** 2018-12-01

**Authors:** Simon W. Davis, Amanda Szymanski, Homa Boms, Thomas Fink, Roberto Cabeza

**Affiliations:** Center for Cognitive Neuroscience, Duke University, Durham, NC, USA; Department of Neurology, Duke University School of Medicine, Durham, NC, USA; Center for Cognitive Neuroscience, Duke University, Durham, NC, USA; Department of Neurology, Duke University School of Medicine, Durham, NC, USA; Center for Cognitive Neuroscience, Duke University, Durham, NC, USA; Center for Cognitive Neuroscience, Duke University, Durham, NC, USA; Center for Cognitive Neuroscience, Duke University, Durham, NC, USA

**Keywords:** Aging, Structural connectivity, Functional connectivity, Episodic memory, Task-related connectivity, SEM

## Abstract

Understanding the precise relation between functional connectivity and structural (white matter) connectivity and how these relationships account for cognitive changes in older adults are major challenges for neuroscience. We investigate these issues using an approach in which structural equation modeling (SEM) is employed to integrate functional and structural connectivity data from younger and older adults (*n* = 62), analyzed with a common framework based on regions connected by canonical tract groups (CTGs). CTGs (e.g., uncinate fasciculus) serve as a common currency between functional and structural connectivity matrices, and ensure equivalent sparsity in connectome information. We used this approach to investigate the neural mechanisms supporting memory for items and memory for associations, and how they are affected by healthy aging. We found that different structural and functional CTGs made independent contributions to source and item memory performance, suggesting that both forms of connectivity underlie age-related differences in specific forms of memory. Furthermore, the relationship between functional and structural connectivity was best explained by a general relationship between latent constructs—a relationship absent in any specific CTG group. These results provide insights into the relationship between structural and functional connectivity patterns, and elucidate their relative contribution to age-related differences in source memory performance.

## Author Summary

Aging is associated with profound changes in how neural systems adapt to perform the same mental operations in youth. Memory functioning, in particular, demonstrates enormous neuroplastic changes in the pattern of distributed, connected networks that enable older adults to perform the same mnemonic operations. However, the relationship between the structural and functional connections supporting these operations is poorly understood. Here we develop a novel algorithm for comparing structural and functional connectivity, and use a comprehensive structural equation model (SEM) to show how these network characteristics contribute to behavioral performance in two forms of episodic memory retrieval. These results suggest that healthy aging is associated with specific ensembles of cooperative contributions from both functional and structural tract groups.

## INTRODUCTION

One of the most consistent patterns in the literature on episodic memory and aging is that older adults tend to be more impaired in episodic memory for associations than in episodic memory for individual items. While this behavioral dissociation has been well known for a long time (Glisky, Polster, & Routhieaux, 1995; Naveh-Benjamin, 2000), cognitive neuroimaging provides a complementary method for investigating the underlying neural mechanisms of this effect (for review, see Old & Naveh-Benjamin, 2008). During the last three decades, cognitive neuroimaging has gradually moved from an emphasis on individual brain regions to a focus on the interactions among brain regions, or connectivity, which can be examined at the functional level using functional MRI (fMRI) and at the structural level using diffusion-weighted imaging (DWI). Given that functional connectivity depends on structural connectivity, a current challenge is how to investigate the relationship between these two forms of connectivity in relation to cognitive function. Here, we propose a new approach for linking structural and functional connectivity data and apply it to the results of an fMRI-DWI study investigating item and source memory in younger and older adults.

There are two main challenges in linking structural and functional connectivity. The first challenge is the problem of *translation* between structural and functional information. Structural matrices are considerably more sparse than functional networks (Wang, Dai, Gong, Zhou, & He, 2015), owing to the fact that while disparate regions may demonstrate (potentially spurious) functional correlations in time course activity, structural connectivity based on diffusion tractography is highly constrained by distance and anatomy (though false positive results are also a signficant issue here; see Maier-Hein et al., 2017). As such, functional connectivity distributions are typically Gaussian, while structural connectivity distributions tend to follow exponential distributions (depending on the metric being used). While the common graph theoretical practice of thresholding and/or binarizing functional and structural connectomes to a common upper threshold (e.g., the top 5% of connections) does help to equalize the amount of graph information contributing information to structure-function comparisons, the underlying sources of information are nonetheless qualitatively distinct in character. Structural connectivity is also static, while functional connectivity is highly dependent on the active process concurrent with data collection (Honey, Kotter, Breakspear, & Sporns, 2007). The second challenge is the problem of the *granularity of mapping*; while a large array of techniques have attempted to delineate structural-functional connectivity relationships at the level of whole-brain parcellations (Betzel et al., 2014; Zimmermann et al., 2016), between discrete pairs of regions (Andrews-Hanna et al., 2007; Davis, Kragel, Madden, & Cabeza, 2012; Dennis et al., 2008), or at the level of voxels (Horn, Ostwald, Reisert, & Blankenburg, 2014), each technique tends to form a unique claim about how the structure-function relationship changes with age. Both of these problems preclude any lasting or satisfying conclusions about how these modalities relate to one another, and have issues unique to datasets that include older adults.

Despite this uncertainty, a number of anatomically defined canonical white matter tracts demonstrate reliable relationships between white matter connectivity and memory, including the fornix, uncinate fasciculus, cingulum, and the genu of the corpus callosum. Multiple measures of connectivity of these tracts have been associated with age-related differences in scores on verbal source memory (Bendlin et al., 2010; Davis et al., 2009; Kennedy & Raz, 2009; Voineskos et al., 2012), spatial- (Oberlin et al., 2016) or object-based source memory (Antonenko et al., 2016), and free recall (Metzler-Baddeley et al., 2012). A number of consistent functional patterns also point to tract-specific relationships in aging and memory, in particular an increase in hippocampal coupling to the prefrontal cortex (PFC) during item memory encoding and retrieval. Thus, while young adults demonstrate connectivity from hippocampus to posterior sensory regions, older adults exhibited greater success-related functional coupling with the dorsolateral PFC and temporal cortex (Dennis et al., 2008; Murty et al., 2009; St. Jacques, Dolcos, & Cabeza, 2009). The consistency of these tract-specific relationships between (a) item memory and ventral temporal pathways and (b) source memory and dorsal frontoparietal pathways suggests a relative specificity of certain connections to specific forms of memory.

A major goal of connectome research is to discover how structural and functional networks in the brain are related—an active area with tremendous interest and wide ramifications in neuroscience. The widespread use of automated connection matrices has led to an explosion of computational solutions to this problem, typically by directly comparing connectivity matrices (Horn et al., 2014), predicting one modality from the other (Abdelnour, Voss, & Raj, 2014; Bowman, Zhang, Derado, & Chen, 2012; Messé, Rudrauf, Giron, & Marrelec, 2015), joint analysis of structural and functional matrices (Honey et al., 2009; Tewarie et al., 2014), or through the comparison of graph-theoretical properties common to structural and functional networks (Betzel et al., 2014; Romero-Garcia, Atienza, & Cantero, 2014). These more data-driven approaches have produced a number of meaningful observations (for an excellent review, see Zhu et al., 2014), principally that the relationship between functional connectivity and structural connectivity also appears to strengthen across the life span, and that this relationship is driven by an increase in the reliance on more long-distance interactions between brain regions (Betzel et al., 2014; Meunier, Stamatakis, & Tyler, 2014). Nonetheless, these computational approaches have largely ignored canonical divisions in the structural anatomy of human white matter pathways. This is problematic in the case of structural models because these models rarely incorporate known anatomy, leading to spurious connections (Maier-Hein et al., 2017), and in the case of functional information these computational solutions rarely take into account the sparsity of structural connection matrices compared with functional data. Thus, finding the adequate basis on which to make the comparison between these modalities is challenging.

The present analysis seeks to address these gaps by using task-based functional connectivity and whole-brain structural connectivity informed by classical white matter anatomy to ask a specific question: *Do functional and structural connectivity make independent contributions to memory in older adults?* Particularly, we explore the possibility that function-structure relationships are best characterized by either specific linkages between modalities for a given tract, or instead reflect a general relationship shared by task-relevant tract groups. We test a model fitting structural and functional connectivity information summarized by canonical tract groups (CTGs) in order to predict source and item memory in younger and older adults. Thus, the structural equation modeling (SEM) approach used here attempts to provide a rigorous statistical framework to examine the complex relationships between age, structural and task-based functional connectivity, and cognitive performance.

## METHODS

### Participants

Seventy-six adults—54 older adults (67.68 ± 6.9 y.o., age range 61–87 y.o.) and 22 younger adults (23.6 ± 3.5 y.o., age range 19–28 y.o.)—participated in the study. All individuals were screened for contraindications to MRI, and seven individuals were excluded because of scanner issues or poor structural or functional imaging quality (see below). Two individuals did not complete the memory task, leaving *N* = 67 with complete data. Written consent was obtained for each participant and they received monetary compensation at the end of the study. All experimental procedures were approved by the Duke University Institutional Review Board.

### Memory Task

#### Materials.

We studied item and source memory using a lexical episodic memory task used in previous studies of our group (Daselaar et al., 2015; Hayes, Buchler, Stokes, Kragel, & Cabeza, 2011). Stimuli consisted of 440 English nouns with normative word frequencies in the lexicon of 5–15 per million, *M* = 8.8 (3.1), and a mean length of *M* = 7.1 (2.3) letters. Unique study and test lists were randomly generated for each participant and words were assigned to the following conditions: item (180 words), source (180), or item lures (80 words—presented only at retrieval as new words). At retrieval, there were four item test lists, each consisting of 45 targets (old words) and 20 lures (nonstudied words), and four source memory test lists, each consisting of 45 studied words.

#### Encoding phase.

Participants studied the words outside the scanner. Words were presented on a computer monitor in white font on a gray background for 3 s with a 1 s interval using Cogent (http://www/vislab.ucl.ac.uk/cogent_2000.php), a stimulus presentation software within MATLAB (https://www.mathworks.com). For half of the trials, participants made a “pleasant/unpleasant” judgment, and a “bigger/smaller than a shoebox” judgment for the other half. Half of the trials were repeated, with the same judgment; however, for the purpose of the present study, we collapsed 1x and 2x encoding trials into one condition for the subsequent fMRI analysis.

#### Retrieval phase.

Approximately 15 min after the encoding phase, participants were tested for their memory of the studied words in the MRI scanner. Words were presented via a mirror in the scanner head coil and a rear projection system using a PC computer running Cogent. There were two retrieval conditions: item memory and source memory. In the item memory retrieval task, participants made new/old responses on a 4-point confidence scale. For the source memory retrieval task, participants were asked to indicate what type of judgment they made earlier on a word on a 4-point scale: definitely pleasant/unpleasant, probably pleasant/unpleasant, probably bigger/smaller, definitely bigger/smaller. Given the current study had no a priori hypotheses about the influence of connectivity measures on confidence, we collapsed across high and low confidence responses. We have clarified this point in the behavioral results. Nonetheless, for a more explicit analysis of confidence measures using this task, see Hayes et al. (2011). Four item and four source memory runs were presented in consecutive blocks to minimize the effects of task switching. Retrieval stimuli were presented for 3 s, with a white crosshair presented for fixation during the intertrial interval (ITI). Stimulus order and ITI jitter (range: 1–7 s) were determined by a genetic algorithm designed to maximize statistical efficiency and facilitate deconvolution of the hemodynamic response (Wager & Nichols, 2003).

### MRI Acquisition and Analysis

**Figure 1.**
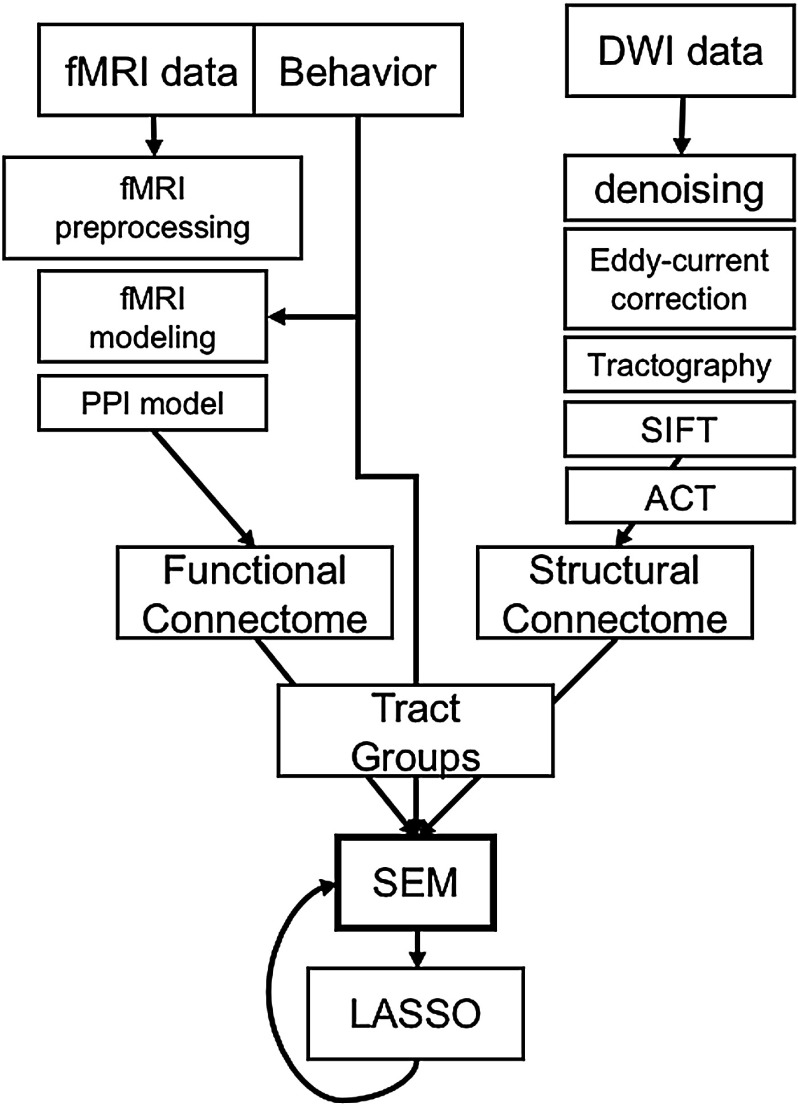
Analytical pipeline. Boxes indicate major steps in the analysis pipeline. Notably, CTGs act as a common currency, or filter, for structural and functional connectome information.

The analytical pipeline is summarized in Figure 1. Participants were first scanned on a 3-T gradient-echo scanner (General Electric 3.0 Tesla Signa Excite HD short bore scanner, equipped with an 8-channel head coil). Coplanar functional images were acquired using an inverse spiral sequence (64 × 64 matrix, time repetition [TR] = 1,700 ms, time echo [TE] = 31 ms, field of view [FOV] 240 mm, 37 slices, 3.8-mm slice thickness, 254 images). Following functional imaging, a high-resolution SPGR series (1-mm sections covering whole brain, interscan spacing = 0, matrix = 256^2^, flip angle = 30, TR = 22 ms, TE = min full, FOV = 19.2 cm) was collected. Finally, DWI data were collected using a single-shot echo-planar imaging sequence (TR = 1,700 ms, slices = 50, thickness = 2.0 mm, FOV = 256 × 256 mm^2^, matrix size = 128 × 128, voxel size = 2 mm^3^, b-value = 1,000 s/mm^2^, diffusion-sensitizing directions = 25, total images = 960, total scan time = 5 min). The anatomical MRI was acquired using a 3-D T1-weighted echo-planar sequence (matrix = 2,562, TR = 12 ms, TE = 5 ms, FOV = 24 cm, slices = 68, slice thickness = 1.9 mm, sections = 248). Scanner noise was reduced with ear plugs, and head motion was minimized with foam pads. Total scan time, including breaks and structural scans, was approximately 1 hr 40 min. Behavioral responses were recorded with a four-key fiber-optic response box (Resonance Technology, Inc.), and when necessary, vision was corrected using MRI-compatible lenses that matched the distance prescription used by the participant.

Preprocessing of functional scans was performed processed using SPM12 (http://www.fil.ion.ucl.ac.uk/spm/software/spm12/). Subjects were excluded on the basis of excessive movement during the fMRI session if any scans demonstrated movement in any x/y/z direction >5 mm, or more than 6% of their total scans with between 3 and 5 mm; based on these criteria, five subjects were removed (two remaining subjects had poor diffusion scans). In all remaining datasets, the first four images were discarded to allow for scanner equilibrium. Images were corrected for asynchronous slice acquisition (slice timing: reference slice = 17, TA = 1.97) and realigned to the first functional image within the series to correct for head motion. For normalization, we used a study-specific template created using unified segmentation and diffeomorphic image registration (DARTEL) in SPM12 (Ashburner, 2007). First, each subject’s image was segmented into gray matter, white matter, and cerebral spinal fluid probabilistic images. The segmented gray matter images were then normalized to MNI space using the DARTEL procedure integrated in SPM12 (Ashburner, 2007), which uses diffeomorphic registration to create a template that is representative of the brain size and shape of all the participants.

#### Construction of connectivity matrices.

Before either structural or functional matrices were constructed, we first sought to establish a consistent parcellation scheme across all subjects and all modalities (DWI, fMRI) that reflects an accurate summary of full connectome effects. Subjects’ T1-weighted image was segmented using the SPM12, yielding a gray matter (GM) and white matter (WM) mask in the T1 native space for each subject. The entire GM was then parcellated into 411 regions of interest (ROIs), each representing a network node by using a version of the Harvard-Oxford Atlas (Tzourio-Mazoyer et al., 2002), subparcellated to obtain a larger number of smaller and more equally sized regional nodes (Fornito, Zalesky, & Bullmore, 2010). The T1-weighted image was then nonlinearly normalized to the ICBM152 template in MNI space using FMRIB’s Nonlinear Image Registration Tool (FNIRT, FSL, https://www.fmrib.ox.ac.uk/fsl/). The inverse transformations were applied to the Harvard-Oxford Atlas in the MNI space, resulting in native-T1-space GM parcellations for each subject. Then, T1-weigted images were coregistered to native diffusion space using the subjects’ unweighted diffusion image (i.e., the b0 image) as a target; this transformation matrix was then applied to the GM parcellations above, using FSL’s FLIRT linear registration tool, resulting in a native-diffusion-space parcellation for each subject.

Structural connection matrices based on DWI data were analyzed utilizing FSL (https://fsl.fmrib.ox.ac.uk/fsl/fslwiki, v5.0.1) and MRtrix (http://mrtrix.org, v3.0) software packages. Data were denoised with MRtrix, corrected with eddy current correction from FSL, and brain extraction was performed with both FSL and MRtrix, whereas bias-field correction was completed with MRtrix. First, fractional anisotropy (FA) maps were created using *dwi2tensor* followed by *tensor2metric* from MRtrix3. Constrained spherical deconvolution (CSD) was utilized in calculating the fiber orientation distribution (FOD). For CSD map generation, the maximum number of spherical harmonic terms was set to 6, and a single-fiber response kernel estimated from white matter voxels with fractional anisotropy FA > 0.3. This FOD was used along with the brain mask to generate whole-brain tractography, with seeding done at random within the mask (Tournier, Calamante, & Connelly, 2007; Tournier, Calamante, Gadian, & Connelly, 2004). Relevant parameters regarding track generation are as follows: seed = at random within mask; step-size = 0.2 mm; 10,000,000 tracts. After tracts were generated, they were filtered using SIFT (spherical-deconvolution informed filtering of tractograms; Smith, Tournier, Calamante, & Connelly, 2012). This process utilizes an algorithm that determines whether a streamline should be removed based on information obtained from the FOD, which improves the selectivity of structural connectomes by using a cost-function to eliminate false positive tracts (Yeh, Smith, Liang, Calamante, & Connelly, 2016). Tracts were “SIFTed” until 1 million tracts remained. In the present analysis we rely on FA (rather than streamlines) as our measure of region-to-region connectivity. FA is high in areas where there is a dense packing of well-myelinated and coherently oriented axonal fibers, and low when axonal structure has been compromised, when fibers are sparse, or when the fibers are organized in a complex geometry that is not adequately captured by the diffusion model. To obtain the mean FA value along each streamline, the output track files were used to sample values along each track, using the FA image as the image to be sampled, resulting in an FA scale file. In turn, connectomes were created by using the original SIFT track files, and the FA scale file from the previous step, with all streamlines in an edge combined into a single scale value based on the mean of those streamlines. FA values were then imputed onto all nonzero pathways; in each structural connection matrix (*A*), the connection strength (*A*_*ij*_) between each pair of cortical regions (_*i*,*j*_) is defined as the average FA value along the fiber tracts connecting these regions. Lack of connections between a pair of regions was set to 0. Thus, the resulting structural connectomes comprised a summary of the fractional anisotropy values along all streamlines connecting a given pair of regions (for other recent studies using similar methods given equivalent DWI scanning parameters, see Qi, Meesters, Nicolay, Ter Haar Romeny, & Ossenblok, 2016; Roberts et al., 2016).

Functional connection matrices representing task-related connection strengths were estimated using a correlational psychophysical interaction (cPPI) analysis (Fornito, Harrison, Zalesky, & Simons, 2012). Briefly, the model relies on the calculation of a PPI regressor for each region, based on the product of that region’s time course and a task regressor of interest, in order to generate a term reflecting the psychophysical interaction between the seed region’s activity and the specified experimental manipulation. In the current study the convolved task regressors for successful (“Hits”) or unsuccessful (“Misses”) retrieval trials were used as the psychological regressor, which coded subsequently remembered and subsequently forgotten word pairs with positive and negative weights, respectively, of equal value. This psychological regressor for successful memory retrieval was based on a linear contrast of Hits > Misses for both source and item memory blocks; new-items trials during the item memory blocks were modeled, but not used in the connectivity analysis. This memory success-related regressor was multiplied with two network time courses for regions *i* and *j*. We then computed the partial correlation *ρ*_*PPI*_*i*_, *PPI*_*j*_ ⋅ *z*_, removing the variance *z* associated with the psychological regressor, the time courses for regions *i* and *j*, and constituent noise regressors. We accounted for the potential effects of head motion and other confounds by assessing the six motion parameters and including these parameters in our partial correlation between regions.

#### Defining tract groups.

We examine only region pairs that are connected by canonical fiber systems, in other words, canonical tract groups (CTGs). This technique affords three main benefits, namely (a) integrating structural and functional connectivity information within a common anatomical framework, (b) constraining the overabundance of functional connections to known anatomy, and (c) simplifying the number of pairwise comparisons in an informed manner. Furthermore, our novel algorithm for summarizing connectivity in canonical, well-characterized fiber systems (and its associated code; see Davis, 2018) is scalable to any of the numerous cortical parcellations (e.g., AAL, Craddock, or even voxelwise parcellations) currently in use. CTG assignment for a given region-to-region connection in the matrix is accomplished by assessing the overlap between the ROIs used to define that matrix. Canonical fiber systems from six tracts defined by the Johns Hopkins University white matter tractography atlas (Hua et al., 2008) include the uncinate fasciculus (UF), inferior fronto-occipital fasciculus (IFOF), forceps minor (FMin), inferior longitudinal fasciculus (ILF), ventral cingulate gyrus (CingHipp), and dorsal cingulate gyrus (Cing), as well as the body of the fornix, based on a novel template (Brown et al., 2017). The corticospinal tract and forceps major were not included because they were not hypothesized to be involved in item or source memory functioning.

**Figure 2.**
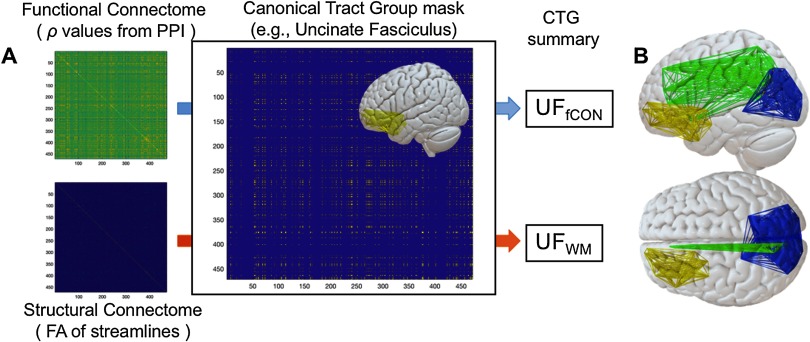
Construction of canonical tract groups. After functional and structural connectome construction, a CTG mask for a particular tract is used to filter functional and structural connectivity information. After functional and structural connectome construction, a CTG mask for a particular tract is used to filter functional and structural connectivity information. All elements in the filtered matrix are then averaged to create a functional and structural estimate for a given CTG (e.g., UF_fCON_ and UF_sCON_) that is amenable to SEM modeling. Also obvious in these images is the relative sparsity of structural connection matrices comparted to functional matrices.

**Figure 3.**
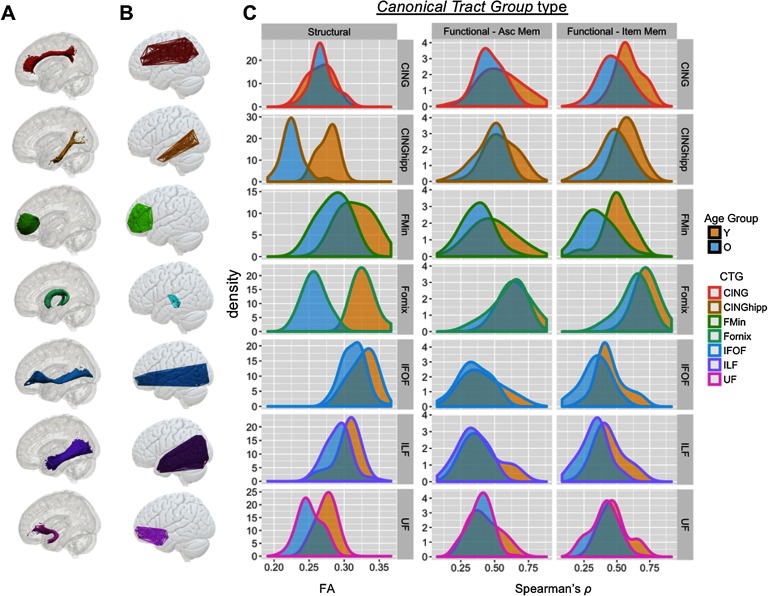
Illustration and age distributions of canonical tract groups. Column (A) describes tract ROIs from the JHU Tract Atlas (see Methods) and column (B) describes the connectome-based canonical tract groups (CTGs) formed from our algorithm, for each tract. (C) Distribution of CTG values for structural connectivity (FA) and functional connectivity (Spearman’s rho, as calculated in PPI) for source and item memory in younger and older adults. CTG values are shown for seven major CTGs that influence the final SEMs below. Age-related statistics are presented in Table 2. Cing = cingulum; CingHip = ventral leg of the cingulum; FMin = forceps minor (or genu); IFOF = inferior fronto-occipital fasciculus; ILF = inferior longitudinal fasciculus; UF = uncinate fasciculus.

The creation of a CTG (e.g., the UF) proceeds in two steps. In the first step, we identify the voxelwise overlap between each tract and each pair of ROIs. If a tract shows overlap with *both* ROI_*A*_ and ROI_*B*_, then we consider the connection between those ROIs as a component of a given CTG (e.g., CTG_UF_, Figure 2). Here we define overlap as 10 voxels, though results are similar with more/less conservative parameters. We refer to this matrix of *n* × *n* elements (where *n* is the number of regions) as the *CTG mask*. This assignment is then repeated for all seven tract groups specified above within the JHU Tract Atlas (FSL). In the second step, functional and structural connectome data for each subject are filtered through these masks, and we calculate a mean of all surviving connectivity values. These values represent inputs to later CFA/SEM models described below. Our analysis based on CTGs offers two clear advantages to data-driven comparisons between these data types: (a) CTGs serve as a common currency between functional and structural connectivity matrices and (b) this method addresses the fact that structural matrices are much more sparse than functional matrices. Thus, by using an identical set of region pairs from the adjacency matrices in each tract group (see individual tract groups in Figure 3), we ensure that the same amount of data contributes to structural or functional connectivity information in the model.

### Structural Equation Modeling

After structural and functional (item and source) matrices are filtered by CTGs and summed across all elements in the matrix, CTG values are averaged across hemisphere for bilateral tracts, and scaled/mean-centered before inclusion into the SEMs (using the R function *scale*). We fit confirmatory SEMs to the mean FA of the seven, bilaterally averaged, WM tract CTGs, which showed different sensitivities to age. These models were used to test the validity of the latent variables for structural connectivity (sCON) based on DWI tractography, and functional connectivity (fCON) associated with successful memory retrieval, based on fMRI collected from either item or source blocks. The full exploratory models for source and item memory combine the structural and functional CFAs in our older adult sample with additional model parameters, including (a) residual covariance between modalities of connectivity information within specific tract groups (e.g., **UF**_***fCON***_—**UF**_***sCON***_), (b) links between the two latent variables (***sCON***—***fCON***), and (c) mutual inputs from these LVs to a behavioral output (either source or item memory accuracy). Model syntax and full model output for full SEMs are available in the GitHub repository mentioned above (Davis, 2018).

Both CFA and full SEMs were fit using the lavaan package (version 0.5, Rosseel, 2012) in R version 3.3.3 (R Development Core Team, 2016), and regularized SEM for complex models using regsem, (version 0.9.2, Jacobucci, Grimm, & McArdle, 2016), which allows the use of LASSO-based regularization while keeping the SEM model intact, adding penalization directly into the estimation of the model. LASSO (least absolute shrinkage and selection operator) imposes a penalty on the regression parameters to ensure that the SEM model remains stable even when the number of predictors is large. Specifically, it uses the L1 norm to apply a LASSO (Tibshirani, 1996) penalty, which enforces sparse solutions by shrinking many regression parameters to 0. We therefore applied LASSO regression to the two full model SEMs in order to penalize the models and reduce the number of contributing CTGs.

Prior to model fitting, variables were scaled to a standard normal distribution. All models were fit using maximum likelihood (ML) estimation using robust standard errors and report overall model fit assessed with the chi-square estimates, root mean squared error of approximation (RMSEA) and its confidence interval, the comparative fit index (CFI). We used the following guidelines for judging good fit (Bagozzi & Yi, 2012): RMSEA below 0.05 (acceptable: 0.05–0.08) and a CFI above 0.97 (acceptable: 0.95–0.97). Model comparison was estimated via the *χ*^2^ likelihood ratio test. The significance of individual paths was tested with *p* values less than 0.05, and the contribution of each predictor was assessed using the *R*^2^ value.

#### Testing age effects using equality constraints.

Lastly, to test the influence of age (for both functional and structural CTGs) in the model, we introduced a younger adult sample into the modeling framework and performed two separate analyses: (a) first, model fit in separate older and younger adult groups using the same likelihood ratio test, fixing parameter estimates derived from the full models above between the two groups; (b) second, in order to explicitly address the differences in SEM model fit between our younger and older adult populations, in order to identify specific parameters in the model affected by age. An equality constraint constrains an SEM model such that, in reaching its solution, it must provide the identical unstandardized coefficient for all parameters within a set that has been designated for equality. Differences in model fit between the constrained and unconstrained model are then assessed with a chi-square difference, indicating whether the freely estimated estimate of a key path in the model differs with age.

## RESULTS

### Behavioral Testing

Source memory accuracy was 0.76 ± 0.016 and RTs were 2.09 s ± 0.49 for successful source trials, and 2.37 s ± 0.58 for unsuccessful source trials. Source memory was greater for items encoded with a *pleasantness* judgment than with a *size* judgment (t_66_ = 5.98, *p* < 0.00004), consistent with previously observed advantages for personal than perceptual source judgments (Dobbins & Wagner, 2005; Naveh-Benjamin, 2000; Naveh-Benjamin & Craik, 1996). Mean hit rates for item memory were 0.85 ± 0.015 and a mean false alarm rate of 0.22 ± 0.008. RTs during the item memory test were 1.59 ± 0.45 for item Hits, and 2.36 for item Misses. Main effects of age were more pronounced for source memory than item memory (F_61,1_ = 12.45, *p* = 0.0008) and overall item memory hit rate (F_61,1_ = 3.68, *p* = 0.04), though the interaction between age group and memory type was not significant (F_61,2_ = 2.18, *p* = 0.14).

### Canonical Tract Groups: Descriptive Statistics and Effects of Age

Following the method outlined above, we developed a semiautomated pipeline for assigning a given connection between ROIs within a standard atlas to a given canonical tract group. While most CTGs showed significant age differences in the structural domain, success-related functional connectivity differences between younger and older adults were far subtler (Table 1, Figure 3). Two-sample *t* tests examining age-related differences in structural and functional connectivity found profound differences in structural connectivity (FA; all *p* < 0.05), and less pronounced age-related difference in functional connectivity (Pearson’s *r*; *p* < 0.05 in 2/7 and 7/7 source or item memory CTGs, respectively; see Table 1 for individual tract statistics). Functional CTGs showed consistently moderate relationships with corresponding structural CTGs (e.g., regions structurally connected by the UF tended to be functionally correlated), even after adjusting for the effects of age (all *r* > 0.21, *p* < 0.05).

**Table 1.** Effects of age on canonical tract groups

**Canonical tract group**	**Z**	Partial correlation with corresponding sCON tract group†
Structural connectivity		
Cing	4.190***	
CingHip	11.959****	
FMin	5.616***	
IFOF	6.746****	
ILF	5.328****	
UF	6.026****	
Fornix	11.889****	

Functional connectivity: Source memory		
Cing	1.791	0.28
CingHip	2.372*	0.31
FMin	1.917*	0.24
IFOF	1.491	0.27
ILF	1.730	0.25
UF	1.368	0.26
Fornix	1.002	0.25

Functional connectivity: Item memory		
Cing	3.737***	0.39
CingHip	3.913***	0.34
FMin	4.353***	0.41
IFOF	3.033**	0.31
ILF	3.415**	0.24
UF	3.043**	0.21
Fornix	2.268*	0.27

† after adjusting for the effects of age. Cing = cingulum; CingHip = ventral leg of the cingulum; FMin = forceps minor (or genu); IFOF = inferior fronto-occipital fasciculus; ILF = inferior longitudinal fasciculus; UF = uncinate fasciculus. Significance: * = *p* < 0.05; ** = *p* < 0.01; *** = *p* < 0.001; **** = *p* < 0.0001.

### SEM Results

#### Confirmatory factor analysis results.

**Figure 4.**
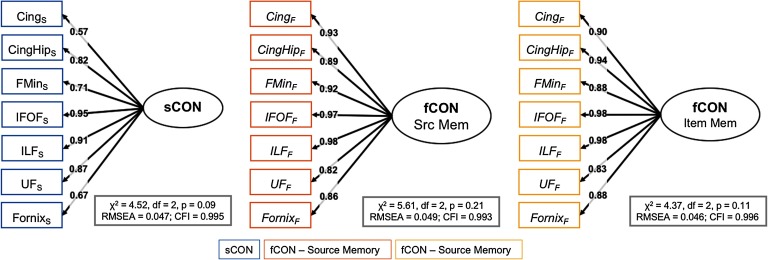
CFA models. Cing = cingulum; CingHip = ventral leg of the cingulum; FMin = forceps minor (or genu); IFOF = inferior-fronto-occipital fasciculus; ILF = inferior longitudinal fasciculus; UF = uncinate fasciculus.

We used SEM to test a range of models of how connectivity supports source memory in older adults. We first examined the reliability of the measures to be used in three confirmatory factor analysis (CFA) models, shown in Figure 4, as well as Table 2. In these models, we hypothesize that two latent variables (functional connectivity or fCON and structural connectivity or sCON) capture the covariance between seven connectivity measures estimated from specific CTGs (described above); while the first factor loading is constrained to 1, every other factor loading is estimated freely. Three single-factor CFA models were evaluated: sCON, fCON_item_, and fCON_source_; these models each fit their respective data well (sCON model: *χ*^2^ = 4.52, *df* = 2, *p* = 0.09, RMSEA = 0.047, CFI = 0.995; fCON_item_ model: *χ*^2^ = 4.37, *df* = 2, *p* = 0.11, RMSEA = 0.046, CFI = 0.996; fCON_source_ model: *χ*^2^ = 5.61, *df* = 2, *p* = 0.21, RMSEA = 0.049, CFI = 0.993), suggesting that structural and functional connectivity can be captured by these single (respectively) dimensions.

**Table 2.** Model fit parameters for three CFA models

**CFA model**	**CTG**	**Parameter estimate**	**Z**	***R*^2^**
White matter model				
	Cing	0.57		0.32
	CingHip	0.82	6.24	0.67
	FMin	0.71	6.07	0.50
	IFOF	0.95	6.38	0.90
	ILF	0.91	6.35	0.82
	UF	0.87	6.26	0.75
	Fornix	0.67	5.97	0.44

Source memory model				
	Cing	0.93		0.86
	CingHip	0.89	9.70	0.79
	FMin	0.92	9.50	0.87
	IFOF	0.97	9.99	0.94
	ILF	0.98	10.03	0.97
	UF	0.82	9.22	0.68
	Fornix	0.86	9.71	0.74

Item memory model				
	Cing	0.90		0.82
	CingHip	0.94	9.71	0.88
	FMin	0.88	9.63	0.77
	IFOF	0.98	9.85	0.95
	ILF	0.98	9.86	0.96
	UF	0.83	9.08	0.69
	Fornix	0.88	9.63	0.77

Parameter estimates are fully standardized. Cing = cingulum; CingHip = ventral leg of the cingulum; FMin = forceps minor (or genu); IFOF = inferior fronto-occipital fasciculus; ILF = inferior longitudinal fasciculus; UF = uncinate fasciculus.

#### Full model results.

Next, using CTGs we fit two full models relating brain connectivity variables to behavioral variables using a standard SEM. These models capture the hypothesis that individual differences in structural and functional connectivity measures make independent contributions to successful memory functioning. These models combine the structural and functional CFAs above by adding (a) residual covariance between modalities for specific tract groups (e.g., ***UF***_***FUNC***_—***UF***_***STRUCT***_), (b) covariance between the two latent variables (***sCON***—***FUNC***), and (c) mutual inputs from these LVs to a behavioral output (either source or item memory accuracy). As the number of tracts, and therefore CTGs, should be linearly correlated, we can ask whether a more parsimonious model shows better fit.

**Figure 5.**
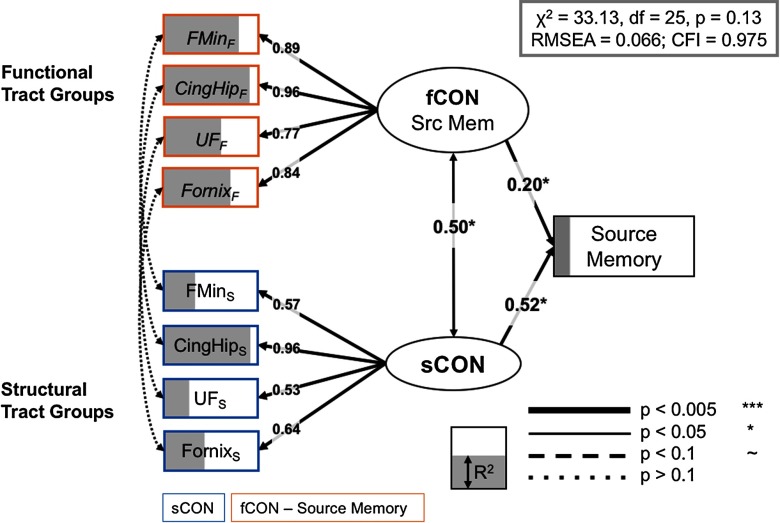
Full model, source memory. Significant paths outlined and *R*^2^ (i.e., the amount of variance accounted for each term in the model) is represented as the degree of shading of the variables. Brain measures only have paths to a corresponding CTG in the other modality, or to the appropriate LV. Notably, no residual covariance modalities for a specific CTG, that is, between functional and structural information (left side of SEM), were significant; indeed only latent constructs for sCON and fCON demonstrated a significant association. CingHip = ventral leg of the cingulum; FMin = forceps minor; UF = uncinate fasciculus.

**Figure 6.**
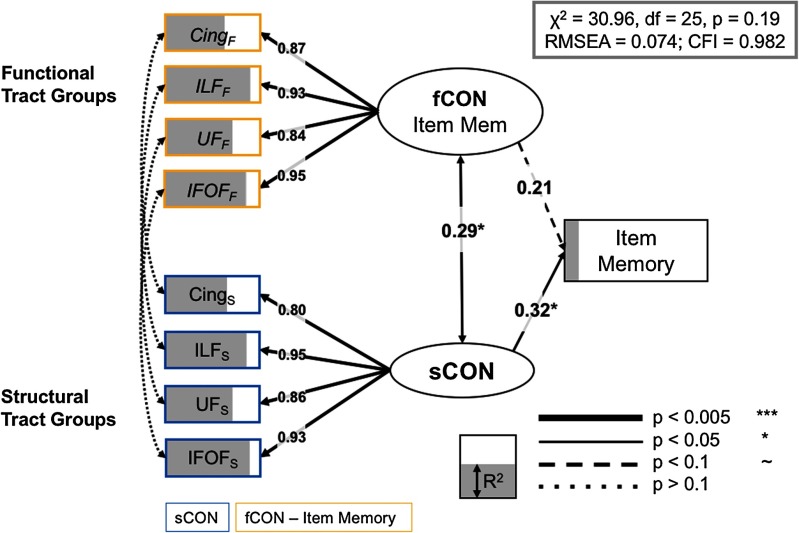
Full model, item memory. Significant paths outlined and *R*^2^ (i.e., the amount of variance accounted for each term in the model) is represented as the degree of shading of the variables. Brain measures only have paths to a corresponding CTG in the other modality, or to the appropriate LV. Notably, just as in the source SEM above, we found no residual covariance between functional and structural information for specific CTGs. Cing = cingulum; IFOF = inferior-fronto-occipital fasciculus; ILF = inferior longitudinal fasciculus; UF = uncinate fasciculus.

We fit two models, one focused on source memory, and one focused on item memory. We first estimated the full models with all seven tract groups, and then estimated the regularized model across a range of lambda values, using RMSEA to compare model fit across each iteration; the best solution for both models was obtained with the fifth lambda value from regularization, (lambda = 0.24, 0.22 for source and item models, respectively), with four tracts that have nonzero weights in the final model for both source and item models. The full regularized model for source memory is shown in Figure 5, and it fits the data quite well: *χ*^2^ = 30.22, *df* = 25, *p* = 0.35, RMSEA = 0.066, CFI = 0.975. The good fit of the full model suggests that the observed covariance pattern in our data is consistent with the statistical constraints imposed by the model, and allows us to further investigate the relations between the cognitive factors and the neural variables. The full model for item memory (Figure 6) also fits the data well: *χ*^2^ = 30.96, *df* = 25, *p* = 0.2, RMSEA = 0.074, CFI = 0.982. While both models share a number of structural and functional inputs, there are a number of unique inputs to each model (discussed below).

#### Tract-specific contributions.

We relied on the LASSO regularization to simplify our model and improve model fit; this technique also implicitly provides a means of identifying the specific tract groups that contribute to memory performance. As noted in the Methods section, we constrained model terms to include both structural and functional information pairs for each CTG, such that we could continue to make explicit hypotheses about structural-functional relationships in our final models. In the source memory model, the uncinate fasciculus, fornix, forceps minor, and hippocampal segment of the cingulum each contributed to the overall model (all *R*^2^ > 0.61/0.35 for structural/functional information, respectively). Furthermore, the inclusion of structural and functional information from the forceps minor of the corpus callosum is in line with previous findings that suggest an important role for prefrontal sCON in episodic memory functioning in older adult populations (Davis et al., 2009; Kennedy & Raz, 2009).

In contrast, the final item memory model relied on paired structural and functional information from the inferior longitudinal fasciculus, the cingulum, the uncinate fasciculus, and the inferior fronto-occipital fasciculus (all *R*^2^ > 0.31/0.34 for structural/functional information, respectively). This result is consistent with the qualitative interpretation that source memory relies on both structural and functional connectivity frontotemporal regions, while item memory shows a greater dependence on structural systems (structural LV std. param. = 0.32, *p* = 0.042) based solely within the temporal lobe (Glisky et al., 1995; Spaniol & Grady, 2012). Lastly, while the latent variable capturing the variance in overall success-related *functional* connectivity did not demonstrate a significant path to behavioral performance on the item memory task (std. param. = 0.21, *p* = 0.18), these functional CTGs nonetheless contributed to the overall model fit; a separate model removing the link from the fCON LV to item memory showed a significant reduction in model fit (Δ*χ*^2^ = 34.22, Δ*df* = 1, *p* < 0.01) Table 3.

**Table 3.** CTG-specific contributions in both item and source memory SEMs

**SEM model**	**Region**	**Structural CTG**		**Functional CTG**	
		***R*^2^**	**Parameter estimate**	***R*^2^**	**Parameter estimate**
Source memory model					
	Latent variable	0.82	0.52**	0.14	0.20*
	CingHip	0.91	0.96****	0.76	0.96****
	FMin	0.62	0.56****	0.51	0.89****
	UF	0.61	0.53***	0.85	0.77****
	Fornix	0.95	0.64****	0.36	0.84****

Item memory model					
	Latent variable	0.39	0.32*	0.34	0.21
	Cing	0.44	0.87****	0.72	0.80****
	ILF	0.64	0.95****	0.67	0.95****
	UF	0.45	0.84****	0.59	0.86****
	IFOF	0.31	0.93****	0.49	0.93****

Parameter estimates are fully standardized. Cing = cingulum; CingHip = ventral leg of the cingulum; FMin = forceps minor (or genu); IFOF = inferior fronto-occipital fasciculus; ILF = inferior longitudinal fasciculus; UF = uncinate fasciculus. Significance: * = *p* < 0.05; ** = *p* < 0.01; *** = *p* < 0.001; **** = *p* < 0.0001.

#### Effects of age.

Our last question was whether the connectivity measures examined herein captured the effect of age on memory; while Table 2 summarizes the influence of age on independent structural and functional connectivity metrics, a more holistic assessment of the role of age in explaining age-related differences in source and item memory may be more adequately characterized within the full SEMs. However, simply combining younger and older adult groups and then comparing full models either with or without age as a covariate would yield unsurprising benefits to including this covariate. This is in fact what we see—the overall fit was significantly worse in a multigroup model in which ages were included, but paths from age to brain factors were fixed at 0, in both the source memory (Δ*χ*^2^ = 89.21, Δ*df* = 2, *p* < 0.001) and item memory models (Δ*χ*^2^ = 66.26, Δ*df* = 2, *p* < 0.005). This result, again unsurprisingly, demonstrates that chronological age captures a significant proportion of variance in the model and has a strong influence on the latent variables for structural and functional connectivity. However, such a multigroup comparison model for young and older adults is nested, and more appropriate techniques may yield more informative results.

An initial assessment of model fit in separate older and younger adult groups (fixing parameter estimates derived from the full models above between the two groups) suggested an overall worse fit in younger than older adults in both source (RMSEA_older_ = 0.066; RMSEA_younger_ = 0.227) and item memory SEMs (RMSEA_older_ = 0.074; RMSEA_younger_ = 0.209). Consequently, we then used the post hoc approach of equality constraints in order to identify specific parameters in the model affected by age. An equality constraint simplifies an SEM model such that, in reaching its solution, it must provide the identical unstandardized coefficient for all parameters within a set that has been designated for equality. In this formulation, the model (i.e., full source and item memory models) is run twice, once without constraints on a particular parameter, and once with the constraint; a significant Δ*χ*^2^ test (with Δ*df*) between these two models therefore suggests that the relevant parameter estimate differs between the groups.

Table 4 summarizes the results of this equality constraint analysis, examining both the change in each input parameter, and—critically—a Δ*χ*^2^ test statistic that characterizes the influence of allowing this parameter to behave unconstrained during model fitting. We found that in our model of source memory, parameters describing structural connectivity in the fornix and functional connectivity in regions connected by the uncinate fasciculus have a stronger influence on model fit in the older adults. In contrast, in our item memory SEM the structural parameters describing sCON in the UF and ILF are generally stronger in the older adult subgroup—a result that is consistent with the fact that the general sCON LV—and not the functional connectivity LV—predicted item memory performance in our model above (Figure 6). This approach is therefore diagnostic of which specific parameters (i.e., which sCON or fCON CTGs) contribute to differences in model fit between younger and older adults. These results suggest an age-related change in the reliance on regions connected by the fornix and UF during source memory retrieval, and the UF and ILF during item memory retrieval.

**Table 4.** Equality constraints analysis results

**SEM model**	**Parameter**	**Δ*χ*^2^**		**Δ in parameter estimate**	
Source memory model					
	Latent variable to:	*sCON*	*fCON*	*sCON*	*fCON*
	CingHip	N/A	N/A		
	FMin	1.23	0.08	0.034	0.066
	UF	0.03	3.44*	0.013	0.108
	Fornix	6.74*	0.01	0.165	0.075

Item memory model					
	Latent variable to:	*sCON*	*fCON*		
	Cing	N/A	N/A		
	ILF	4.73*	0.25	0.094	0.019
	UF	7.15**	1.33	0.135	0.078
	IFOF	0.37	1.74	0.022	0.049

Parameter estimates are fully standardized. Cing = cingulum; CingHip = ventral leg of the cingulum; FMin = forceps minor (or genu); IFOF = inferior fronto-occipital fasciculus; ILF = inferior longitudinal fasciculus; UF = uncinate fasciculus. Significance: * = *p* < 0.05; ** = *p* < 0.01.

## DISCUSSION

By combining multiple behavioral, demographic, and brain measures from a large sample of younger and older adults, we provide evidence that age-related differences in source and item memory are dissociable by their functional and structural connectivity profiles. In our best-fitting model, individual CTGs based on canonical fiber systems make independent contributions to both forms of memory. First, we found that the relationship between structural and functional connectivity information was best characterized by an intermediate level of relationship. Although no *specific* CTGs demonstrated a significant association between their corresponding fCON and sCON values (i.e., fCON–sCON covariance, as indicated by the curved, dotted lines in Figures 5 and 6), a more general sCON–fCON relationship between latent variables built on these tract-specific measurements was significant in both source and item memory SEMs, suggesting a more general relationship between structural and functional modalities. Second, we found that both sCON and fCON make independent contributions to source memory performance, while only sCON influenced behavior in the item memory SEM. Lastly, age-related influences on our model were much stronger for sCON than for fCON, but age was an essential component of the full model. Our results therefore demonstrate that age-related declines in memory are unlikely to be driven by a single fiber system or a single data type, but emerge as a confluence of functional and structural differences in multiple anatomically connected systems.

### Structure-Function Relationships

Evidence has shown that brain topology (i.e., structure) supports fluid dynamics (i.e., function), and that brain dynamics in turn reinforce structure via synaptic plasticity. In a very influential work, Honey et al. (2007) showed that this relationship is highly dependent on the characteristics of the functional data used to test this relationship, including the timescale, local clustering, and brain state. Our use of CTGs integrates structural and functional connectivity information within a common anatomical framework, achieved by constraining functional connections to known anatomy. More specifically, summarizing the structural (FA based on tractography streamlines) and functional (Spearman’s rho based on task-related PPI) relationships between pairs of regions connected by canonical tract groups (e.g., the uncinate fasciculus) helps to link empirical results obtained via adjacency matrices—a now common basis for most graph-theoretical approaches to characterizing aging brain networks—with clinically minded approaches centered on canonical fiber systems.

In our best fitting model for source or item memory, no residual covariance between modalities (i.e., structural and functional connectivity) for the same CTG reached significance, while latent variables for structural and functional connectivity did show a significant association. When the LV-LV pathway was set to 0, model fit significantly decreased (Δ*χ*^2^ = 9.78, Δ*df* = 1, *p* < 0.016). It is worthwhile to note that, outside the SEM framework, functional and structural CTGs were reliably correlated across subjects (Table 1; all but one CTG *r* > 0.21, even after adjusting for age). Taken together, these results suggest that the relationship between structural and functional connectivity estimates may be best characterized on an intermediate level. Many of the age-related differences to white matter may appear to manifest as global changes across different major white matter tracts (Penke et al., 2010), and driven by causal factors that affect white matter, such as small vessel disease, myelin depletion, or iron accumulation. Nonetheless, a growing model-based literature is emerging that suggests that a more constrained set of critical white matter fiber systems (forceps minor, cingulum, uncinate fasciculus) provide the best fit for models seeking to explain age-related differences in attention, memory, and processing speed (Kievit et al., 2016; Lovden et al., 2013; Voineskos et al., 2012).

### Tract-Specific Effects on Source and Item Memory

Whether tract-specific relationships or general global declines in white matter health best predict age-related declines in executive and mnemonic performance is a matter of some ongoing debate (Kievit et al., 2016, 2014; Lovden et al., 2013; Penke et al., 2010); the value of such debates rests on the anatomical specificity used in creating structural and functional connectivity values to predict age-related differences in behavior. Our result suggests an *intermediate* conclusion to the general versus specific debate: While the residual covariance between specific structural-functional modalities for a specific fiber tract does not drive the success of a model of source or item memory, there are nonetheless a subset of specific tract groups that provide the best fit to these data. We found that the structural and functional connectivity based on the fornix had a selective positive influence for source, but not item memory, consistent with theoretical and empirical results supporting the role of this structure in source retrieval (Aggleton & Brown, 1999; Antonenko et al., 2016). The fornix is a key white matter tract of the medial temporal lobe memory system, interconnecting the hippocampal formation with subcortical structures in the basal forebrain and diencephalon. There is evidence of altered white matter microstructure in the fornix in healthy older adults (Antonenko et al., 2016; Persson et al., 2006), and measures of fornix microstructure may be useful in detecting early/preclinical Alzheimer’s disease stages (Nowrangi & Rosenberg, 2015). Similarly, the finding that the uncinate fasciculus—which connects anterior temporal and inferior frontal cortices—is implicated in both our source and item memory SEMs is consistent with evidence linking this tract to age-related decline in memory functioning across a wide array of tasks, including visual object location (Metzler-Baddeley, Jones, Belaroussi, Aggleton, & O’Sullivan, 2011), color-picture associations (Lockhart et al., 2012), working memory (Burzynska et al., 2013), and verbal learning (Lancaster et al., 2016). Similarly, the item memory–specific role of the IFOF fits well with electrostimulation-based studies that have shown semantic paraphasias in response to (disruptive) stimulation of this fiber system (Duffau et al., 2005).

Interestingly, we found that the genu, or forceps minor of the corpus callosum (FMin in our models), which connects left and right prefrontal cortex, contributed significantly to source but not to item memory, a finding that is consistent with the assumption that source memory is more dependent on PFC-mediated functions (Shimamura, 1995), and that bilateral PFC activity may serve a compensatory role in age-related decline (Cabeza, 2002). We and others have found that the FA of the genu predicts behavior on a range of episodic memory (Davis et al., 2009; Henson et al., 2016) and executive function tasks (Kievit et al., 2016, 2014) in elderly populations. The dissociation in source versus item memory performance (Glisky et al., 1995) is consistent with abundant evidence that the PFC is more critical for source than item memory performance in aging populations (Duarte, Ranganath, Trujillo, & Knight, 2006; Leshikar & Duarte, 2014; Old & Naveh-Benjamin, 2008; Spaniol & Grady, 2012), and reliance on structural and functional connectivity in the PFC may be a means of counteracting observed source deficits (Dennis et al., 2008; Naveh-Benjamin, Hussain, Guez, & Bar-On, 2003).

### Effects of Age

Our last findings focused on age-related differences between younger and older adults: First, estimates of model fit (e.g., RMSEA) were generally better in older than younger adults; second, modeling equality constraints for all parameters suggested that structural connectivity of the fornix and functional connectivity mediated by the UF show the strongest differences between younger and older adults in source memory. Model comparison in aging samples is an unresolved issue, and no unanimity exists on how to include chronological age as an operative term in SEMs examining brain and behavioral factors (however, see Bender, Volkle, & Raz, 2016; Henson et al., 2016; Kievit et al., 2016). Nonetheless, consensus opinions are emerging about the role of cross-sectional and longitudinal datasets in estimating the effects of “age” versus the effects of “aging.” While our cross-sectional sample is unable to draw inferences on “aging,” we nonetheless take advantage of the specific qualities of our sample to evaluate how well our model describes younger and older adult groups separately, in order to draw inferences on the age-specificity of the observed effects.

Age effects on structural connectivity revealed with DWI are regionally diverse and typically show an anterior-to-posterior gradient of age-related decline (Davis et al., 2009; Head et al., 2004; Sullivan, Adalsteinsson, & Pfefferbaum, 2006). Consistent with this evidence, we observed strong declines in FA across nearly all structural connectivity groups, or CTGs (Table 2). While a number of studies have found single correlation (Kennedy & Raz, 2009) or mediation patterns (Madden et al., 2010; Oberlin et al., 2016) that help to explain how these white matter structures mediated cognitive decline, our analysis advances on these approaches by considering all of these regions simultaneously, within a statistically rigorous framework. We found that within older adults, successful source memory was associated with structural and functional connectivity in a set of canonical fiber tracts connecting either within or between frontal regions: the forceps minor, UF, CingHip, and fornix (Figure 5), even when all brain measures were adjusted for chronological age. In contrast, an overlapping but more ventral set of regions, including the inferior longitudinal fasciculus, helped to predict successful item memory performance (Figure 6). Furthermore, estimates of the influence of age on our model are difficult to estimate within this SEM framework, but nonetheless suggest an important role for (a) structural integrity of the fornix, and functional information conveyed by regions connected by the UF in predicting source memory differences between older and younger adults, and (b) structural information in nearly all CTGs in item memory, given the significant Δ*χ*^2^ after these single parameters were allowed to be freely estimated (holding all other parameters constant).

With respect to functional connectivity, the finding that CTGs connecting either within (forceps minor) or between the PFC and other regions (UF, IFOF) explain significant variance in source memory scores is consistent with the general observation that older adults show higher levels of PFC activity across a range of cognitive tasks (Grady, 2012). Our findings of age-related increases in task-related frontotemporal functional connectivity during successful source memory provide evidence for a compensatory mechanism by demonstrating a reliance on PFC connectivity from three major inferior temporal white matter fibers: the FMin, UF, and fornix. Our connectivity measure was based on task-related data, and furthermore used an explicit contrast between Hits > Misses, in order to isolate connectivity related to successful memory functioning. Our result is also consistent with a handful of studies that have identified an increased reliance on frontotemporal connectivity to maintain source memory function (Dennis et al., 2008; Spaniol & Grady, 2012), and this finding more generally supports the idea that frontotemporal interactions support source memory performance (Backus, Schoffelen, Szebenyi, Hanslmayr, & Doeller, 2016).

Lastly, we must acknowledge some of the relevant limitations of our approach, with respect to modeling the influence of functional and structural connectivity in the aging brain and its influence on different forms of memory. Clearly a larger, more balanced sample may have helped to improve some of the inferences concerning the relative influence of connectivity factors in promoting age-related differences in source and item memory, and a more distributed sample across a range of ages may allow for alternative modeling strategies for this particular application of SEM. The growth of large population-representative datasets with this kind of information (e.g., Cam-CAN.org) is heartening in this respect. Furthermore, in order to establish a more robust model of the relationship between structural and functional dynamics, it would be necessary to follow people over time and establish what causal factors (critical developmental periods, nutrition, cardiovascular fitness, etc.) influence this relationship.

## CONCLUSIONS

To summarize, we have applied a novel connectome summary algorithm to show that age-related differences in source and item memory are dependent on distinct combinations of structural and functional connectivity tract groups. These results help to link graph analyses of structural and functional data in anatomically informed and theory-driven manner. Usually, an implicit assumption is that if the structure of the network is observable, an inference of the underlying structure of the connected system can be based on diffusion tractography structure. Our results test this assumption explicitly, by using an analytical method that puts structural and functional connectivity information on equal footing. These results provide further insights into the interplay between structural and functional connectivity patterns and help to elucidate their relative contribution to age-related differences in source memory performance.

## AUTHOR CONTRIBUTIONS

Simon W. Davis: Conceptualization; Data curation; Formal analysis; Investigation; Methodology; Software; Visualization; Writing – original draft; Writing – review & editing. Amanda Szymanski: Data curation; Formal analysis; Writing – original draft; Writing – review & editing. Homa Boms: Data curation; Formal analysis. Thomas Fink: Data curation; Formal analysis. Roberto Cabeza: Conceptualization; Funding acquisition; Resources; Supervision; Writing – original draft; Writing – review & editing.

## FUNDING INFORMATION

Roberto Cabeza, National Institute on Aging (http://dx.doi.org/10.13039/100000049), Award ID: R01AG19731. Simon W. Davis, National Institute on Aging (http://dx.doi.org/10.13039/100000049), Award ID: K01AG053539.

## TECHNICAL TERMS

Functional connectivity: Coherent functional activity between different cortical regions, over the course of a certain temporal range.
Structural connectivity: White matter connectivity between different cortical regions, typically assessed with the number of white matter streamlines.
Source memory: Memory for the specific context in which an item is learned, typically reported with the who/what/when of the context.
Canonical white matter tract: A macroanatomical brain feature of white matter anatomy with a well-known course and connectivity.
Item memory: Memory for the item itself, typically reported with an old/new decision.
CTG (canonical tract group): Our derived measure describing the regions connected by a canonical fiber (or tract) bundle (e.g., uncinate fasciculus, forceps minor).
SEM (structural equation modeling): A diverse array of statistical methods used to assess unobservable latent constructs using observable data.
FA (fractional anisotropy): A value between zero and one that describes the degree of anisotropy of a diffusion process.
LASSO (least absolute shrinkage and selection operator): A common method of regularization used to efficiently estimate a parsimonious model.
Model fit: The ability of an overidentified model to reproduce the correlation or covariance matrix of the variables.
Parameter estimates: Constants that describe the size of the relationship between any pair of observed or unobserved variables in the model.
